# Clinical study on low-level laser therapy to control postoperative pain and quality of life in patients undergoing root canal treatment

**DOI:** 10.6026/973206300220615

**Published:** 2026-02-28

**Authors:** Chavva Lakshmi Charan Reddy, Rashmi Patil, Taniya Maqsood, Siddiq Ahmed, Priyadarshani, Azeem Sultana, Md Kafeel Ahmed

**Affiliations:** 1 Department of Conservative Dentistry and Endodontics, Panineeya Institute of Dental Sciences and Research Centre, Hyderabad, Telangana, India; 2 Department of Conservative Dentistry and Endodontics, P.M.N.M Dental College and Hospital, Bagalkot, Karnataka, India; 3 Department of Conservative Dentistry and Endodontics, Private Consultant in Hyderabad, Telangana, India; 4 Department of Conservative Dental Sciences, Ibn Sina National College for Medical Studies, Jeddah, Saudi Arabia; 5 Department of Conservative Dentistry and Endodontics, Bhabha College of Dental Sciences, Bhopal, Madhya Pradesh, India; 6 Deparment of Conservative Dentistry and Endodontics, Government Dental College and Hospital, Hyderabad, Telangana, India; 7 Department of Periodontology and Implantology, MNR Dental College and Hospital, Sangareddy, Hyderabad, Telangana, India

**Keywords:** Low-level laser therapy, root canal treatment, postoperative pain, quality of life, visual analog scale, oral health impact profile

## Abstract

Postoperative pain affects 25-40% of Randomized Controlled Trial (RCT) patients, impairing oral health-related quality of life (OHRQoL). This double-blind RCT randomized 80 single-visit RCT patients to low-level laser therapy (LLLT) (808 nm periapical laser) or placebo postoperatively. LLLT reduced 24-hour Visual Analog Scale (VAS) pain scores (2.1±1.2 vs 4.8±1.5; p<0.001) across all timepoints. Oral Health Impact Profile-14 (OHIP-14) scores improved significantly (15.3±3.1 vs 22.7±4.5; p<0.001) with 58% less analgesic use. Immediate LLLT advances RCT recovery as safe adjunct for pain control and enhanced OHRQoL.

## Background:

Root canal treatment (RCT) is one of the basic procedures in endodontics for the treatment of pathologies of the pulp and periapical area with rates reported as over 85% [1]. Despite the progress in instrumentation and obturation techniques, postoperative pain is a common sequela and is observed in 25 - 40% [2]. This inflammatory response is caused by mechanical, chemical and microbial irritation of periapical tissues during canal preparation, which results in the release of inflammatory mediators such as prostaglandins, bradykinin and substance P [3]. The resultant pain significantly compromises oral health-related quality of life (OHRQoL) leading to masticatory dysfunction, sleep disturbance and psychological distress [4]. Conventional pain management depends on systemic analgesics, mainly the non-steroidal anti-inflammatory drugs (NSAIDs), which are associated with the risks of gastrointestinal ulceration, renal toxicity and cardiovascular problems [5]. Consequently, there has been a great deal of interest in adjunctive, non-pharmacological interventions. Low-level laser therapy (LLLT) also known as photobiomodulation therapy uses low-intensity coherent light to modulate cellular processes via cytochrome c oxidase activation and increased adenosine triphosphate synthesis [6]. This mechanism dampens down inflammatory cascades, edema and also modulates nociceptor activity and exerts analgesic effects [7]. Recent studies have been able to show the efficacy of LLLT in postoperative pain reduction in several types of dental procedures, such as third molar extraction and periodontal surgery [8]. However, there is still limited and heterogeneous evidence specific to RCT. A 2017 systematic review found only 3 RCTs assessing the efficacy of LLLT in endodontics, concluding that although the initial data was encouraging, discrepancies in the methods and small sample sizes prevented authors from making conclusive recommendations [9]. Of interest, most of the current studies have mainly concerned the evaluation of pain intensity as a main outcome, while there has been little or no attention given to patient-centered outcomes such as OHRQoL. Furthermore, optimal LLLT parameters, including wavelength, energy density and application protocol are still empirically arrived at, rather than evidence based [10]. The Oral Health Impact Profile-14 (OHIP-14) is a validated tool for measuring OHRQoL and contains seven domains: functional limitation, physical pain, psychological discomfort, physical disability, psychological disability, social disability and handicap [11]. Therefore, it is of interest to assess the effectivity of adjunctive LLLT in the control of postoperative pain and at enhancing OHRQoL in patients undergoing RCT on single rooted teeth on a single visit.

## Materials and Methods:

## Study design and setting:

This prospective, double blind, randomized, placebo controlled clinical trial, was conducted at the Department of Conservative Dentistry and Endodontists between January 2023 and June 2024.

## Sample size calculation:

Sample size was calculated on the basis of a pilot study of 20 patients in which a mean difference in 24-hour VAS scores between groups of patients was 2.5 points (standard deviation 2.0). Using G*Power software version 3.1.9.7, with a=0.05, power=0.80 and effect size=1.25, the minimum needed sample was 34 patients per group. To for account 15% attrition, 80 patients were recruited.

## Participant selection:

Eighty Adult patients (18-65 years) with primary RCT in single root teeth with vital pulps showing symptomatic irreversible pulpitis were taken for study. Inclusion criteria were: ASA physical status of I-II, preoperative pain (VAS = 5), absence of periapical radiolucency, ability to understand study protocols. Exclusion criteria included: pregnancy or lactation, systemic diseases (diabetes, immunological disorders), the use of analgesic/anti-inflammatory drugs within 24 hours pre-operatively, periodontal pockets >4 mm and previous RCT on the same tooth as well as not allowing the patient to come for follow up appointments.

## Randomization and blinding:

Participants were randomly assigned to LLLT (n=40) or placebo (n=40) using computer generated block randomization (block size 4). Allocation concealment was ensured by the use of sequentially numbered, opaque, sealed envelopes. Patients and the outcome assessor were masked to which group they were in. Treating clinician was not masked for laser operation. The placebo group had the same probe placement without laser emission.

## Intervention protocol:

All the procedures were carried out by one experienced endodontist. Following local anesthesia (2% lidocaine with 1:80,000 epinephrins), rubber dam isolation and access cavity preparation, root canals were instrumented with ProTaper Gold rotary files (Dentsply Sirona) and 2.5% sodium hypochlorite irrigation. Canals were obturated using gutta percha and AH plus sealer using single cone technique. In the LLLT group, a diode laser (Doctor Smile, Lambda Scientific, 808 nm wavelength, 100 mW output power) was used in continuous mode at the periapical region for 40 seconds (4 J/cm^2^ energy density) using a sterile tip of a fiberoptic (diameter 400 um). The tip was placed intraorally on the buccal mucosa corresponding to the apex of the root. The placebo group had the tips placed identically for 40 seconds without emission of laser. All patients were given instructions after surgery and a standard analgesic regimen (ibuprofen 400 mg every 6 hours as needed).

## Outcome measures:

Primary outcomes were pain intensity postoperatively assessed with a 10-point VAS (0=no pain, 10=worst imaginable pain) at 6, 12, 24, 48 hours and 7 days. Secondary outcomes included OHRQoL measured using OHIP-14 questionnaire at 24 hours and 7 days and total analgesic tablet consumption measured using patient diaries. Baseline demographic data, preoperative pain scores and pulpal diagnosis were recorded.

## Statistical analysis:

Data were analyzed in the software, version 26.0, of the Statistical Package for Social Sciences (SPSS). Normality was determined using Shapiro-Wilk test. Independent t-test was used to compare continuous variables between groups and Mann-Wilcoxon U test was used for non-parametric data. Repeated measures Analysis of Variance (ANOVA) was used to analyze VAS scores over time. Chi-square test was used to compare the categorical variables. Statistical significance was fixed at p<0.05. Results were presented as mean+standard deviation (SD) or median (interquartile range).

## Results:

Eighty patients completed the study (40 per group) with no dropouts. Baseline demographic and clinical characteristics were comparable between groups (p>0.05) (Table 1). The mean age was 38.6±11.2 years in the LLLT group and 37.9±10.8 years in placebo. Preoperative VAS scores were 4.2±1.1 and 4.1±1.3, respectively (p=0.721). Postoperative pain analysis revealed significantly lower VAS scores in the LLLT group at all time intervals (p<0.001). Pain scores peaked at 12 hours in both groups, with the LLLT group exhibiting 53% lower intensity (2.9±1.1 vs 6.1±1.4). At 24 hours, mean VAS was 2.1±1.2 in the LLLT group compared to 4.8±1.5 in placebo (p<0.001). By day 7, pain scores were negligible in both groups but remained significantly different (0.3±0.5 vs 0.8±0.7, p=0.002) (Table 2). OHRQoL assessment demonstrated significant improvements in the LLLT group. At 24 hours, total OHIP-14 scores were 15.3±3.1 versus 22.7±4.5 in placebo (p<0.001), representing a 33% improvement. All seven OHIP domains showed significant intergroup differences, with physical pain and psychological discomfort showing largest effect sizes. At 7 days, scores decreased in both groups but remained significantly better in the LLLT cohort (5.2±2.1 vs 8.9±3.3, p<0.001) (Table 3). Analgesic consumption was significantly lower in the LLLT group, with mean tablet intake of 2.8±1.4 compared to 6.7±2.1 in placebo (p<0.001), representing a 58% reduction. Only 70% of LLLT patients required any analgesia versus 95% in the placebo group (p=0.003). No adverse events or complications were reported in either group.

## Discussion:

The main result of this randomized controlled trial shows that the adjunctive application of LLLT immediately after single visit RCT is significantly reducing pain intensity, analgesic consumption by 58% and significantly increasing the OHRQoL in comparison with placebo treatment. These results would support our hypothesis and provide strong evidence for incorporating LLLT into the standard endodontic protocols. The observed pain reduction is consistent with mechanistic understanding of the photobiomodulation of LLLT. At the cellular level, the irradiation of 808 nm laser triggers the cytochrome c oxidase located in the mitochondria, improving the generation of ATP and the generation of reactive oxygen species [12]. This cascade blocks the expression of cyclooxygenase-2 and the synthesis of prostaglandin E2 and reduces inflammatory hyperalgesia [13]. Furthermore, LLLT inhibits pro-inflammatory cytokines such as interleukin-1v and tumor necrosis factor-and increases anti-inflammatory mediators [14]. The energy density of 4 J/cm^2^ used in this study falls within the therapeutic window reported by Naseri *et al.* [15], who demonstrated significant postoperative pain reduction after root canal treatment using LLLT parameters including 4 J/cm^2^ energy density. Our VAS results are temporally concordant with common patterns of pain after root canal treatments with a peak time of 12 hours and slow resolution by day 7. The 53% reduction in pain at 12 hours and sustained effect through 48 hours confirms the durability of analgesic benefit from LLLT, aligning with Guerreiro *et al.* [16], who reported VAS reductions of 1.5-2 points at 24 hours using LLLT post-RCT. Conversely, Ismail *et al.* [17] showed no significant difference using 940 nm LLLT (1.2 J/cm^2^), likely due to suboptimal energy density. The significant OHRQoL improvement is a new contribution in the endodontic literature. While earlier research had a narrow focus on pain scores, with our integration of OHIP-14 we have captured the multi-faced impact of postoperative discomfort. The 33% improvement at 24 hours, especially in the physical pain and psychological discomfort areas, highlights the capacity of LLLT to maintain daily functioning. This finding is in accord with the broader dental literature that has linked acute pain to transient OHRQoL deterioration [4]. The continuous improvement at day 7 despite the diminishing pain scores lead to the possibility that LLLT may be speeding up the overall recovery perception beyond the simple reduction of nociceptive perception. Decreased use of analgesics has important clinical and economic implications. The 58% decrease in intake of ibuprofen to reduce the risks for adverse events associated with NSAids such as gastrointestinal haemorrhage and renal impairment is of particular importance in elderly or medically compromised patients [5]. From a public health standpoint, reduced pharmaceutical dependency is in line with the current trends of minimally invasive, biologically-based interventions. This pharmacological sparing effect is in agreement with the results from periodontal surgery studies, in which LLLT was able to reduce postoperative analgesic requirement by 40-60% [8]. Methodological strengths are used to strengthen our conclusions. The double-blind, placebo- controlled design reduced bias and standardized RCT protocols ensured homogeneity of procedures. Strict inclusion criteria eliminated confounders like periapical pathologies and systemic pathologies and isolated the impacts of LLLT on iatrogenic inflammation. The 100% retention rate is an indication of the feasibility of the protocol as well as patient acceptance. However, limitations lead to recognition. The single center recruitment may result in limited generalizability and follow-up duration in our study captured only acute pain which may miss the subacute or chronic developments. Additionally, the 808nm wavelength and 4J/cm^2^ parameters, whilst evidence-based, may not be universal best practices in various anatomical regions or phenotypes of patients. Future studies should investigate the efficacy of LLLT in teeth with multiple roots, in which the complex anatomy may affect the results. Dose-response studies of different energy density and application timing could optimize treatment protocols. Genetic polymorphisms of inflammatory responses are another frontier; interleukin-1 genotyping may be useful to identify super-responders [18]. Furthermore, cost-effective analyses of the costs of LLLT implementation relative to savings in analgesics expenditures as well as increased productivity would enhance support for clinical adoption of this strategy. In conclusion, this trial has provided strong evidence that adjunctive LLLT is a significant benefit in improving postoperative pain control and OHRQoL after RCT whilst reducing pharmacological dependency. These results provide an argument for a routine inclusion of LLLT in endodontic practice, pending validation by multi-centre trials and health economic analyses.

## Conclusion:

We show the significant benefit of single application LLLT and the therapy was found to be safe, well tolerated and clinically efficacious at all-time points measured. These findings support the use of LLLT as a useful non-pharmacological adjunct treatment in modern endodontics which offers greater patient-centered outcomes beyond conventional RCT protocols. The incorporation of LLLT by clinicians should be considered in optimizing postoperative recovery and minimizing pharmaceutical dependency.

## Figures and Tables

**Table 1:** Demographic and baseline clinical characteristics of study participants

**Characteristic**	**LLLT Group (n=40)**	**Placebo Group (n=40)**	**p-value**
Age (years), Mean±SD	38.6±11.2	37.9±10.8	0.768
Gender, n (%)			0.823
Male	19 (47.5)	18 (45.0)	
Female	21 (52.5)	22 (55.0)	
Tooth type, n (%)			0.912
Central incisor	8 (20.0)	9 (22.5)	
Lateral incisor	7 (17.5)	6 (15.0)	
Canine	10 (25.0)	11 (27.5)	
Premolar	15 (37.5)	14 (35.0)	
Preoperative VAS, Mean±SD	4.2±1.1	4.1±1.3	0.721
VAS: Visual Analog Scale;
SD: Standard Deviation

**Table 2:** Postoperative VAS scores at different time intervals

**Time Point**	**LLLT Group (n=40)**	**Placebo Group (n=40)**	**Mean Difference (95% CI)**	**p-value**
6 hours	2.3±0.9	4.5±1.2	2.2 (1.7, 2.7)	<0.001
12 hours	2.9±1.1	6.1±1.4	3.2 (2.6, 3.8)	<0.001
24 hours	2.1±1.2	4.8±1.5	2.7 (2.1, 3.3)	<0.001
48 hours	1.4±0.8	3.2±1.1	1.8 (1.3, 2.3)	<0.001
7 days	0.3±0.5	0.8±0.7	0.5 (0.2, 0.8)	0.002
Values are mean ± SD; VAS:
Visual Analog Scale (0-10);
CI: Confidence Interval

**Table 3:** OHIP-14 scores and analgesic consumption

**Outcome Measure**	**LLLT Group (n=40)**	**Placebo Group (n=40)**	**p-value**
OHIP-14 at 24 hours, Mean±SD	15.3±3.1	22.7±4.5	<0.001
OHIP-14 at 7 days, Mean±SD	5.2±2.1	8.9±3.3	<0.001
Analgesic tablets (total), Mean±SD	2.8±1.4	6.7±2.1	<0.001
Patients requiring analgesics, n (%)	28 (70.0)	38 (95.0)	0.003
OHIP-14: Oral health impact
profile-14 (range 0-56)
